# Reconstruction of extensive gluteal defect following excision of hidradenitis suppurativa using a combined lumbar artery perforator and split superior gluteus maximus musculocutaneous flap: A case report

**DOI:** 10.1016/j.jpra.2026.05.053

**Published:** 2026-06-07

**Authors:** Keisuke Okabe, Noriko Aramaki-Hattori, Tatsuyuki Ishii, Kazuo Kishi

**Affiliations:** Department of Plastic and Reconstructive Surgery, School of Medicine, Keio University, 35, Shinanomachi, Shinjuku-ku, Tokyo, Japan

**Keywords:** Gluteus maximus musculocutaneous flap, Lumbar artery perforator flap, Hidradenitis suppurativa, Gluteal reconstruction

## Abstract

Hidradenitis suppurativa is a chronic, inflammatory, recurrent, debilitating, skin follicular disease characterized by painful nodules and recurrent persistent drainage. Severe hidradenitis suppurativa not only reduces quality of life but also serves as a precursor to squamous cell carcinoma and can cause sepsis; therefore, complete excision of the lesion is recommended. We report a case of extensive hidradenitis suppurativa affecting most of the bilateral buttocks. Since it was determined that the extensive lesion in this case could not be covered in a single stage using any known single flap, we decided to perform reconstruction using a newly devised combined flap. For the area cephalad of the iliac crest, a large rotation flap centered on a lumbar artery perforator was designed. For the area caudal to the iliac crest, the superficial layer of the cephalic portion of the gluteus maximus muscle was split, and a musculocutaneous flap was designed to be elevated to include the perforating branch of the superior gluteal artery. These flaps were elevated as a single combined flap. Postoperatively, the flap blood flow was stable and the graft took well. There was no longer any persistent discharge from the lesion, and no functional impairment was observed following the elevation of the gluteus maximus musculocutaneous flap. Reconstruction using a combined lumbar artery perforator and split superior gluteus maximus musculocutaneous flap was considered a useful method, as the technique is simple and can cover extensive gluteal defects.

## Introduction

Hidradenitis suppurativa (HS) is a skin disease characterized by recurrent and chronic painful indurations and abscesses in areas rich in apocrine sweat glands, such as the axilla, groin, and buttocks. The primary causes are believed to be blocked hair follicles and immune system abnormalities, with smoking and obesity also contributing factors.^1^ HS is highly debilitating and severely impairs the psychological well-being and quality of life of patients.^1^ Surgical treatment involving complete excision of the affected area is effective for HS, but subsequent reconstruction can be challenging. While skin grafting has been reported for defect repair^2^, it often results in cosmetic deformity and pain due to lack of cushioning, making flap reconstruction preferable. Here we report a case of reconstruction using a combined flap consisting of a lumbar artery perforator flap and a split superior gluteus maximus musculocutaneous flap for extensive bilateral HS of the buttocks.

## Case report

**Fig. 1:**
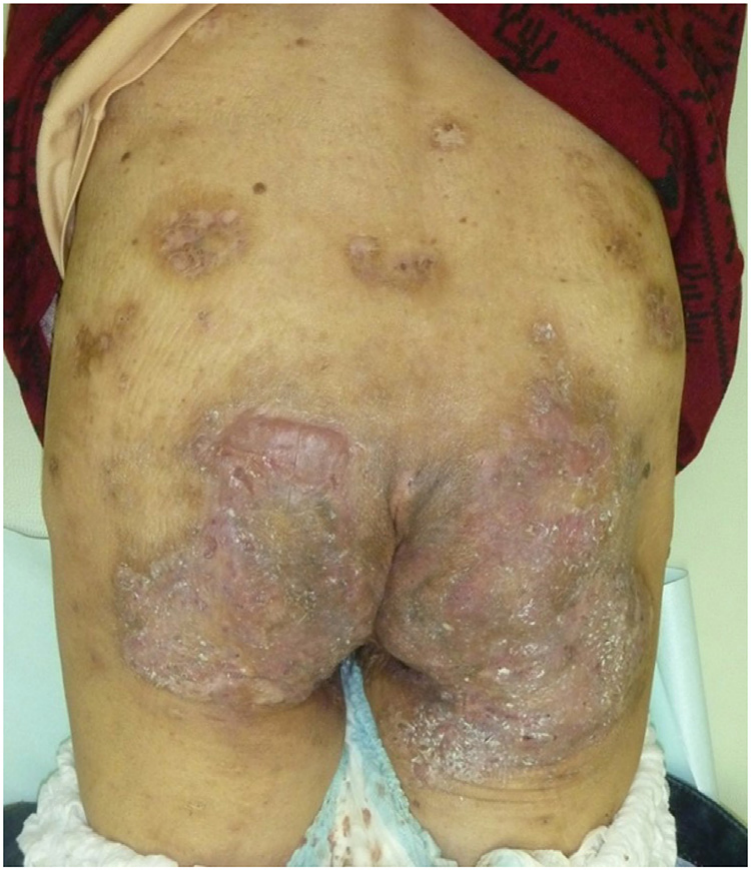
A 54-year-old woman presenting with extensive hidradenitis suppurativa affecting both buttocks. Indurations measuring 15 × 20 cm and 15 × 15 cm for right and left gluteal regions were noted. There was persistent purulent discharge from multiple fistulas.

A 54-year-old female patient presented with extensive induration and chronic suppuration in both buttocks. Since her youth, she developed fistulas on both buttocks that discharged pus, and the affected area gradually expanded. Due to persistent discharge staining her clothes, she always wore diapers. She had a smoking history of over 30 years and had diabetes as a comorbid condition. On initial examination, extensive indurations were palpable over bilateral buttocks. Persistent purulent discharge was observed from multiple fistula tracts (Fig. 1).

**Fig. 2:**
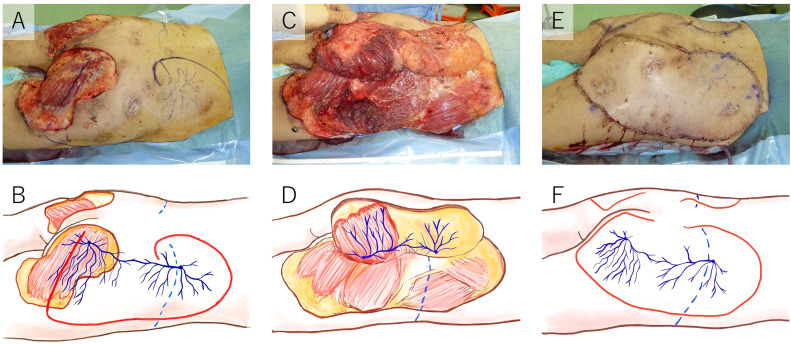
Reconstruction using a combined lumbar artery perforator and split superior gluteal maximus musculocutaneous flap. (A, B) Flap design. The positions of the lumbar artery perforator and the superior gluteal artery perforator were confirmed using Doppler ultrasound prior to surgery. (C, D) Intraoperative findings. The flap was elevated with tissue attached around the lumbar artery perforator for several centimeters. The superficial layer of the gluteus maximus was split to half its thickness and elevated from the distal end to four-fifths of its length, while the proximal one-fifth was left intact. As a result, the superficial branch of the superior gluteal artery perforator was included within the flap. (E, F) Postoperative condition with the defect covered by the flaps.

We planned to resect the entire HS lesion and perform immediate reconstruction using a local flap. The estimated defect size was 15 × 20 cm on the right buttock and 15 × 15 cm on the left buttock; given that the patient was a thin, petite woman, a near-total gluteal defect was anticipated. It was considered impossible to cover the defect in a single stage using a single known flap. We devised a plan to create a combined flap incorporating the superior gluteal artery perforator and the lumbar artery perforator, utilizing their close connection, and to cover the defect by moving a large amount of tissue from the cephalic side. The positions of the lumbar artery perforator and the superior gluteal artery perforator were confirmed using Doppler ultrasound prior to surgery. For the area cephalad of the iliac crest, a large rotation flap centered on the preoperatively marked lumbar artery perforator was designed. The flap was elevated with tissue attached around the perforating artery for several centimeters, allowing it to be easily moved into the defect site without strain. For the area caudal to the iliac crest, the superficial layer of the cephalic portion of the gluteus maximus muscle was split using the method previously reported.^3^ Briefly, the superficial layer of the gluteus maximus was split to half its thickness and elevated from the distal end to four-fifths of its length, while the proximal one-fifth was left intact. As a result, the superficial branch of the superior gluteal artery perforator was included within the flap. These flaps were elevated as a single combined flap (Fig. 2). After moving the flap to the defect site, indocyanine green (ICG) was administered intravenously, and flap blood flow was observed using a photodynamic eye (pde-neo®, Hamamatsu photonics). The ICG fluorescence appeared from locations corresponding to the lumbar artery perforator and superior gluteal artery perforator, and rapidly spread throughout the entire flap (Supplementary Video 1).

**Fig. 3:**
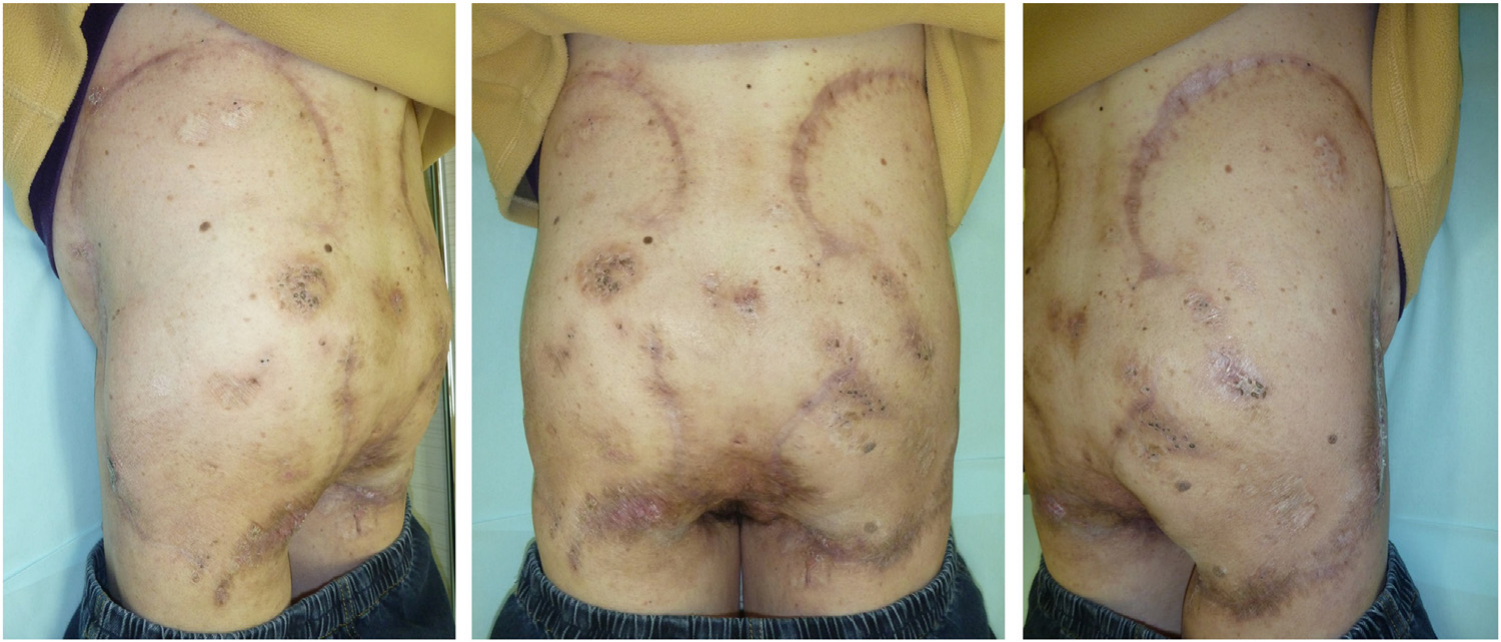
Findings at 10 months postoperatively. The flap showed no signs of ischemia or congestion and has fully taken. Purulent discharge has ceased, and the patient's quality of life has significantly improved.

Postoperatively, the flap did not develop congestion or ischemia. However, minor wound dehiscence was observed at two sites—the right greater trochanter and the left gluteal cleft—one week after surgery. Conservative management resulted in epithelialization and healing within three weeks. At the 10-month postoperative follow-up, there was no sign of suppuration (Fig. 3). No impairment in walking or motor function was observed postoperatively. She no longer needed to wear diapers constantly as she had before surgery, and her quality of life improved.

## Discussion

HS is a skin disease characterized by recurrent and chronic painful indurations and abscesses in areas rich in apocrine sweat glands, such as the axilla, groin, and buttocks.^1^ HS carries a risk of developing squamous cell carcinoma^4^ and can also pose a life-threatening danger due to severe infection.^5^ Therefore, surgical treatment involving complete excision of the affected area is recommended for patients with prolonged duration and severe conditions. We previously reported on a reconstruction method, named extended split superior gluteus maximus musculocutaneous (ESS-GM-MC) flap, that expands the flap area and involves splitting and elevating the gluteus maximus muscle, based on our research using cadaver vascular dissection studies.^6^ Although the GM muscle has been used without much concern for functional deficits, use of the entire muscle in ambulatory patients creates some functional problems. On the contrary, if either the upper or lower half of the GM muscle is used, no disability is caused in an ambulatory patient.^7^ This method is technically simple and is considered particularly useful for reconstructing lesions in the lower buttocks. In the present case, since the lesion of HS extended cranially and medially, this method alone was considered insufficient for filling the defect. Similarly, it was considered impossible to cover the defect, including the donor site, using known single flaps such as the superior gluteal artery perforator flap or the lumbar artery perforator flap. Therefore, a combined approach using a split superior gluteus maximus musculocutaneous flap and a lumbo-gluteal flap was employed for this patient. The lumbo-gluteal flap was clinically applied based on anatomical findings and reported by Nakajima et al. in 1984^8^; since then, it has become widely used as a lumbar artery perforator flap.^9^ Anatomically, the superior gluteal artery perforator and lumbar artery perforator have been reported to have close anastomotic connections.^6^^,^^10^ Intraoperative assessment of flap blood flow using ICG also demonstrated that these two perforators are closely anastomosed with one another (Supplementary Video 1). By elevating these two flaps as a combined flap, it was possible to reconstruct the patient's extensive tissue defect in a single stage. Reconstruction using a combined lumbar artery perforator and split superior gluteus maximus musculocutaneous flap was considered a useful reconstructive method, as the technique is simple and can cover extensive defects.

## Ethical approval

Not required.

## Conflicts of interest

None declared.

## Appendix Supplementary materials

Image, application 1

Image, application 2
