# Supplementary material for: Reconstruction of extensive gluteal defect following excision of hidradenitis suppurativa using a combined lumbar artery perforator and split superior gluteus maximus musculocutaneous flap: A case report
Source: JPRAS Open. 2026 Jun 7;51:76–81. doi: 10.1016/j.jpra.2026.05.053 (PMC13316296; doi:10.1016/j.jpra.2026.05.053)
Supplement: Supplementary file 1 [file mmc1.docx]

**Supplementary Movie 1**. Evaluation of flap blood flow by photodynamic eye (pde-neo®, Hamamatsu Photonics) after indocyanine green (ICG) administration. The ICG fluorescence appeared from locations corresponding to the lumbar artery perforator and superior gluteal artery perforator, and rapidly spread throughout the entire flap
